# Impact of Massachusetts law prohibiting flavored tobacco products sales on cross-border cigarette sales

**DOI:** 10.1371/journal.pone.0274022

**Published:** 2022-09-13

**Authors:** Fatma Romeh M. Ali, Brian A. King, Elizabeth L. Seaman, Donna Vallone, Barbara Schillo

**Affiliations:** 1 Non-Infectious Disease Programs, CDC Foundation, Atlanta, Georgia, United States of America; 2 CDC, Atlanta, Georgia, United States of America; 3 Truth Initiative, Washington, DC, United States of America; Icahn School of Medicine at Mount Sinai, UNITED STATES

## Abstract

**Background:**

In June 2020, Massachusetts implemented a law prohibiting the sale of all flavored tobacco products, including menthol cigarettes. This law was associated with significant declines in overall cigarette and menthol cigarette sales in Massachusetts, however it is unknown whether the law has increased cross-border sales in neighboring states where menthol cigarettes are still sold.

**Methods:**

U.S. cigarette retail scanner data were licensed from the IRi Company. Cigarette pack sales were summed in 4-week periods during January 2020-December 2021 (n = 832). Outcomes were state-level pack sales per 1000 population, overall and by flavor status (menthol and non-flavored). A difference-in-differences analysis was used to examine adjusted sales for Massachusetts border states (New Hampshire, Connecticut, Vermont, and Rhode Island) before (January 2020-May 2020) and after (June 2020-December 2021) the Massachusetts’s law, compared to 28 non-border states. Control variables included state and time fixed effects; real price per pack; tobacco control policies; COVID-19 cases and deaths, and related statewide closure; and state sociodemographic characteristics.

**Results:**

Following the law, unadjusted sales of menthol, non-flavored, and overall cigarettes trended upward in border states; however, these increases were not statistically significant or different from sales patterns in non-border states. This finding persisted after accounting for product prices, tobacco control policies, the COVID-19 pandemic, sociodemographic factors, and fixed effects.

**Conclusion:**

Laws prohibiting the sale of flavored tobacco products, including menthol products, reduce access to these products, while having no significant impact on cross-border sales in neighboring states where menthol cigarettes are sold.

## Introduction

In June 2020, Massachusetts implemented a law prohibiting all flavored tobacco sales, including menthol cigarettes [1]. As of March 2022, Massachusetts is the only state to prohibit the sale of menthol cigarettes. A recent study found that the law was associated with declines in overall cigarette and menthol cigarette sales statewide [2]. Similarly, a Massachusetts government report found cigarette tax revenue declined statewide during 2021–2022; the same report also found cigarette tax revenue increased in some neighboring states [3]. In contrast, a recent study has shown that overall tobacco sales decreased in most neighboring states following the Massachusetts law [4]. The impact of the Massachusetts law on cigarette sales in bordering states, controlling for potential confounding factors, is unknown. Therefore, this study assessed menthol and non-flavored cigarette sales in states bordering Massachusetts before and after the law compared to non-border states, using a multivariate difference-in-differences analysis controlling for potential confounding factors.

## Methods

### Data source

U.S. cigarette retail scanner data were licensed from the IRi Company, which reflect retail scanner data from convenience stores, gas stations, grocery stores, drugstores/pharmacies, mass merchandiser outlets, club stores, dollar stores, and military outlets, but does not include online sales. Out of 50 states, data from three states (Alaska, Hawaii, and Montana) were not commercially available. In addition, data from eight states (Delaware, Idaho, Kansas, Minnesota, Mississippi, Nebraska, New Jersey, and New Mexico) were excluded because their data did not include convenience stores, which represented 99.6% of national e-cigarette sales in 2020 based on IRI data. Therefore, IRi was assessed to provide reliable data for 38 states, other than Massachusetts. The analysis focused on states that did not impose state or local restrictions on menthol cigarette sales to disentangle the effect of Massachusetts law from other policies. This resulted in six states, including New York, California, Colorado, Illinois, Maine, and Oregon, being excluded from the analysis because some localities in these states restricted menthol cigarette sales. The list of border states included the following four states: New Hampshire, Connecticut, Vermont, and Rhode Island. The list of non-border states (comparison states) included the remaining 28 states: Alabama, Arizona, Arkansas, Florida, Georgia, Indiana, Iowa, Kentucky, Louisiana, Maryland, Michigan, Missouri, Nevada, North Carolina, North Dakota, Ohio, Oklahoma, Pennsylvania, South Carolina, South Dakota, Tennessee, Texas, Utah, Virginia, Washington, West Virginia, Wisconsin, Wyoming.

We restricted the analysis to the period after state e-cigarette flavor bans in late 2019 to tease out changes in these policies and their potential effects on menthol cigarette sales. The final sample included 832 four-week sales of cigarette data consisting of 104 observations from Massachusetts border states (20 from before and 84 from after the menthol flavor ban) and 728 observations from the non-border comparison states (140 from before and 558 from after the menthol flavor ban). Sensitivity analyses including the six excluded states with local menthol policies and periods before year 2020 yielded similar results (Table 2).

### Statistical analysis

The following difference-in-differences (DID) model (Eq 1) was used to examine adjusted sales for Massachusetts border states (New Hampshire, Connecticut, Vermont, and Rhode Island) before (January 2020-May 2020) and after (June 2020-December 2021) the Massachusetts’s law, compared to 28 non-border states.

$${\text{Cigarette}\;\text{Sales}}_{st}=\beta_{0}+\beta_{1}MABan_{st}+\beta_{3}Price_{st}+\alpha X_{st}+\delta_{s}+\delta_{t}+\varepsilon_{st}$$ (1)

where Cigarette Sales_*st*_ is 4-week pack sales of menthol, non-flavored, and all cigarettes per 1000 people in state *s* at time *t*. Cigarette units were standardized so that each pack equals 20 cigarettes. *MABan*_*st*_ represents border states after Massachusetts’ flavor ban, equals to 1 for border states after May 2020 and 0 otherwise. *Price*_*st*_ is weighted mean of real price per pack in state *s* at time *t*, which was calculated as total 4-week inflation adjusted dollar sales divided by total 4-week pack sales. ***X***_*st*_ is a vector of state-level time-varying variables, which included cigarette excise taxes [5], the percentage of state population covered by comprehensive smoke-free air laws [6, 7], tobacco control funding as a percentage of CDC recommended funding level [8], and state sociodemographic factors (population percentages by race/ethnicity and age group [9], median annual household income [10], and monthly unemployment rates [11]). Population percentages by race/ethnicity and age group and median annual household income were not available for year 2021. State growth rates in these variables during 2019–2020 were used to estimate the values in year 2021. Furthermore, recent research has shown that adult smokers increased cigarette smoking during COVID-19 pandemic in order to deal with pandemic-related anxiety and despite facing finical difficulties, smokers made efforts to ensure their cigarette supply [12]. Because COVID-19 might have affected states differently, and therefore might not be fully captured in time fixed effects, we controlled for COVID-19 impact measures, including cumulative numbers of COVID-19 cases and deaths per 1000 population [13], dates of statewide closure due to COVID-19 and reopening dates [14]. *δ*_*s*_ is state fixed effects to account for any unobservable state-level factors that do not vary over time. *δ*_*t*_ is time fixed effects to account for any unobservable time-variant factors that are common across all states.

Linear regression models were used to estimate the DID models and standard errors were clustered within states. *P* < .05 was considered statistically significant. Analyses were conducted in Stata (version17; StataCorp, College Station, TX). Advarra Institutional Review Board (IRB) determined this research does not involve human subject participation and did not require IRB oversight.

### Parallel-trends test of DID models

**Fig 1:**
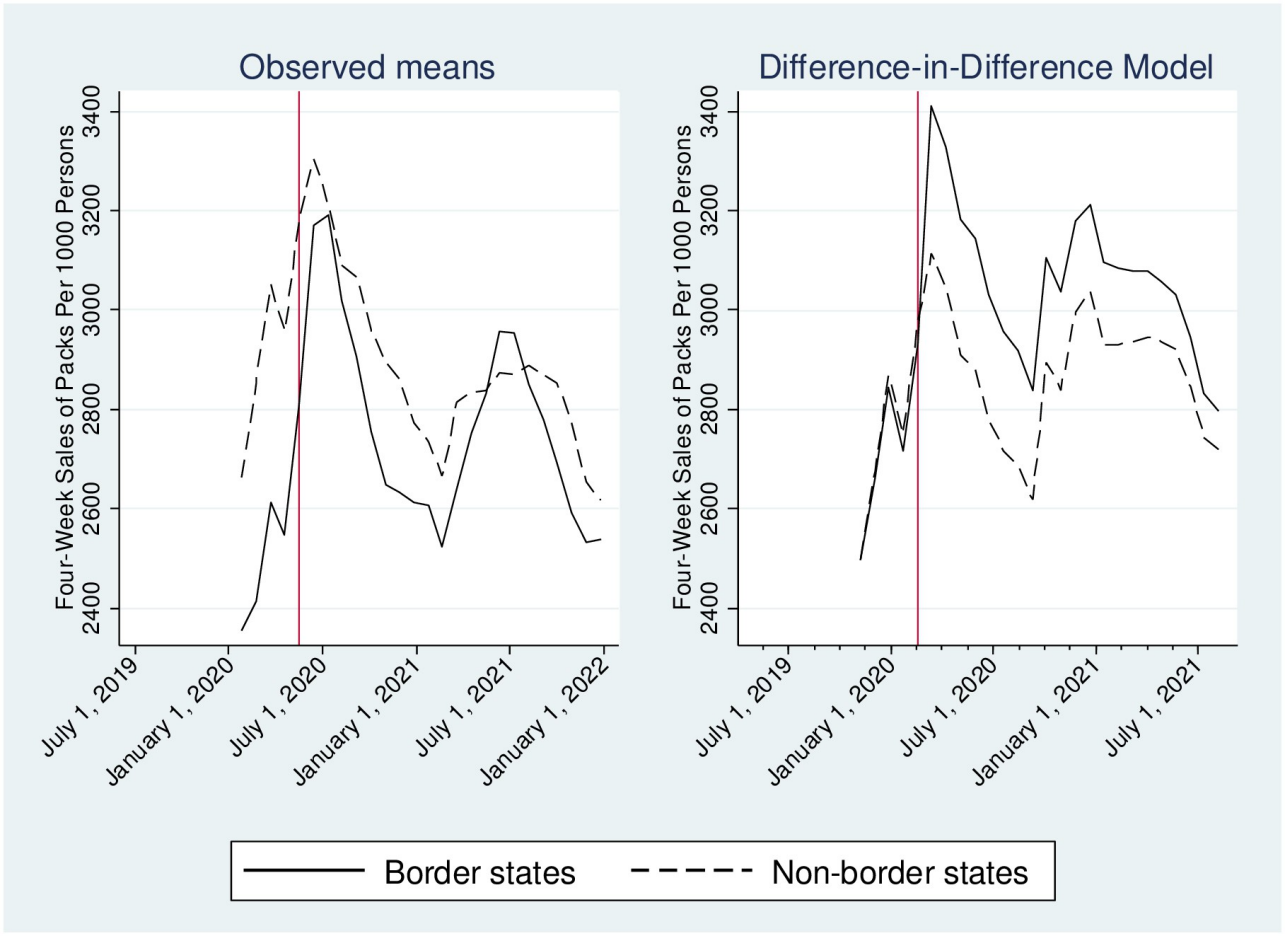
Graphical diagnostics for parallel trends assumption using both observed means and difference-in-difference estimates^a^. ^a^ See the text for a description of the difference-in-difference estimated model. Retail sales data were obtained from Information Resources, Inc. (IRI) for convenience stores, gas stations, grocery stores, drugstores/pharmacies, mass merchandiser outlets, club stores, dollar stores, and military sales. Unit sales were standardized by one pack of 20 cigarettes. The list of border states included New Hampshire, Connecticut, Vermont, and Rhode Island. The list of non-border states included the following 28 states: Alabama, Arizona, Arkansas, Florida, Georgia, Indiana, Iowa, Kentucky, Louisiana, Maryland, Michigan, Missouri, Nevada, North Carolina, North Dakota, Ohio, Oklahoma, Pennsylvania, South Carolina, South Dakota, Tennessee, Texas, Utah, Virginia, Washington, West Virginia, Wisconsin, Wyoming. Each bar in the figure represents a 4-week sum of cigarette sales, averaged across products and across states within each state group.

One of the assumptions of the DID model is that cigarette sales trends of border states were parallel to non-border states prior to the Massachusetts law. We performed a test of the parallel trends assumption by augmenting the DID model with two interaction terms that capture the differences in slopes between border and non-border states in pre- and post- Massachusetts law. The first was an interaction between a linear time trend, an indicator for border states, and an indicator for the time period before the Massachusetts law. The second was an interaction between a linear time trend, an indicator for border states, and an indicator for the time period after the Massachusetts law. The coefficient of the first interaction term was statistically insignificant for menthol, non-flavored, and all cigarette sales, and therefore we have no evidence to reject the parallel-trends test assumption. Fig 1 provides a graphical diagnostic for parallel trends, which supports the parallel-trends assumption.

## Results

Table 1 presents means of all covariates for Massachusetts, as well as both border and non-border states during January 2020-May 2020 (i.e., before the Massachusetts law). The percentage of Black persons was 10.4% in Massachusetts, compared to 7.2% and 14.3% in border and non-border states, respectively. Massachusetts had higher household annual median income but had a greater burden of COVID-19 outcomes than other states. Comparing border versus non-border states, border states had higher household annual median income, higher cigarette tax, and higher cigarette price compared to non-border states. However, border states, had a greater burden of COVID-19 outcomes than non-border states.

**Table 1:** Means of state characteristics for border and non-border states during January 2020-May 2020 (before Massachusetts law prohibiting flavored tobacco products sales).

Variable	Border states^a^	Non-border states^b^	Massachusetts
*Age*			
% Population under 18 years	19.00	22.55	19.46
% Population 18–24	9.74	9.24	9.92
% Population 25–44	24.75	26.18	26.92
% Population 45–64	27.45	24.84	26.31
% Population ≥64	19.06	17.18	17.38
*Race*			
White	87.32	78.64	80.21
Black	7.15	14.32	10.41
American Indian and Alaska Native	1.33	2.66	1.08
Asian	3.99	4.02	8.07
Native Hawaiian and Other Pacific Islander	0.20	0.36	0.23
Unemployment Rate (%)	4.85	4.36	4.44
Household annual median income ($)	79602.25	65398.00	87764.00
*COVID-19*			
Monthly cumulative total cases per 1000 persons	1.81	0.88	3.55
Monthly cumulative total deaths per 1000 persons	0.11	0.04	0.24
State is closed in a given month (%)	45.00	34.29	40.00
Duration of closure (in days)	26.35	16.79	30.40
Weighted cigarette price per pack	9.21	6.40	10.28
cigarette tax ($)	3.37	1.31	3.51
% Population covered by smoke free air laws	50.00	46.03	100.00
% Tobacco control funding from CDC recommended level	9.33	16.44	6.90

^a^ The list of border states of Massachusetts included New Hampshire, Connecticut, Vermont, and Rhode Island.

^b^The list of non-border states of Massachusetts included the following 28 states: Alabama, Arizona, Arkansas, Florida, Georgia, Indiana, Iowa, Kentucky, Louisiana, Maryland, Michigan, Missouri, Nevada, North Carolina, North Dakota, Ohio, Oklahoma, Pennsylvania, South Carolina, South Dakota, Tennessee, Texas, Utah, Virginia, Washington, West Virginia, Wisconsin, Wyoming.

**Fig 2:**
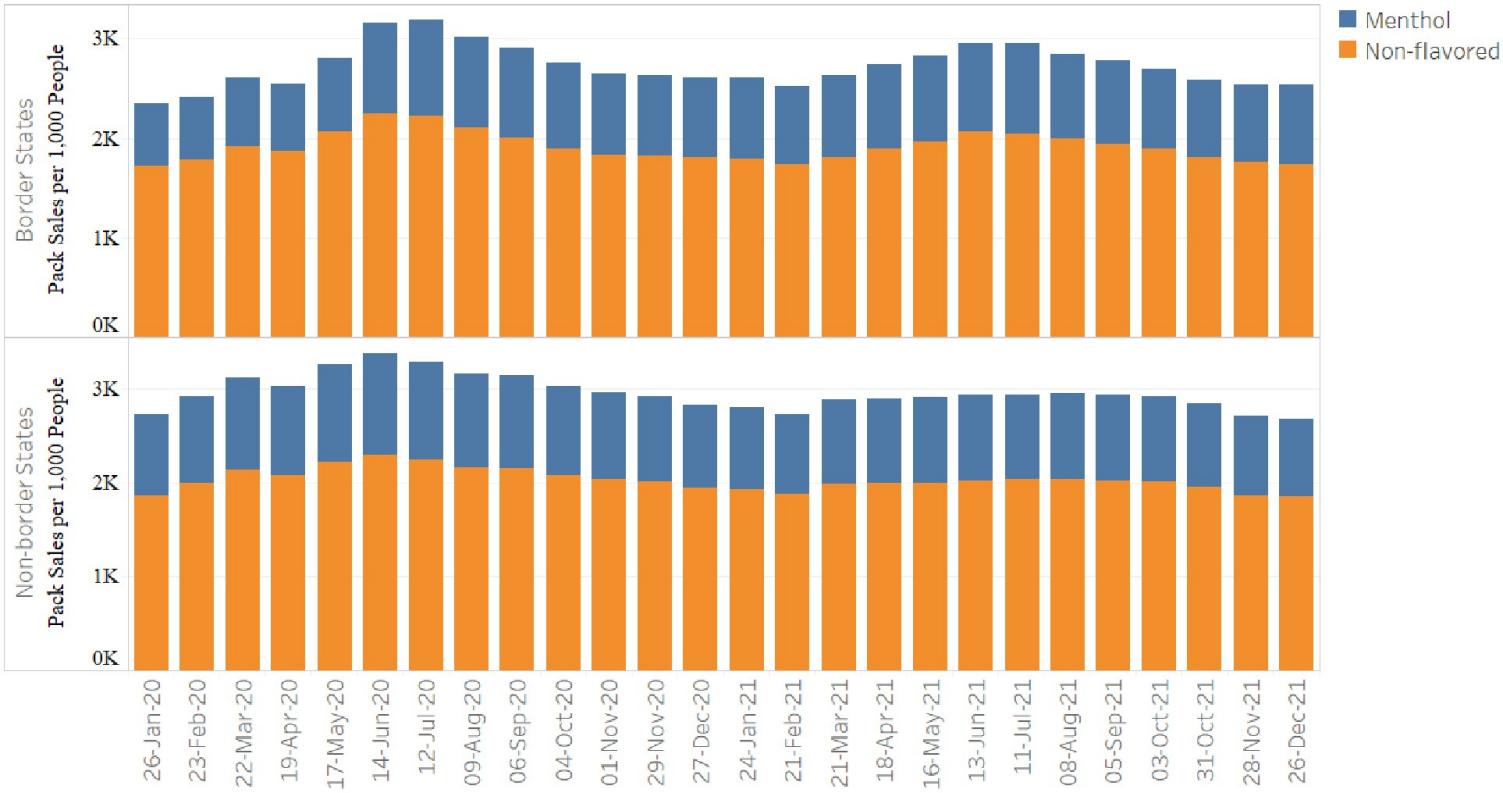
Sales of cigarette packs,^a^ by flavor, in border versus non-border states,^b^ January 2020-December 2021^c^. ^a^Retail sales data were obtained from Information Resources, Inc. (IRI) for convenience stores, gas stations, grocery stores, drugstores/pharmacies, mass merchandiser outlets, club stores, dollar stores, and military sales. Unit sales were standardized by one pack of 20 cigarettes. ^b^ The list of border states included New Hampshire, Connecticut, Vermont, and Rhode Island. The list of non-border states included the following 28 states: Alabama, Arizona, Arkansas, Florida, Georgia, Indiana, Iowa, Kentucky, Louisiana, Maryland, Michigan, Missouri, Nevada, North Carolina, North Dakota, Ohio, Oklahoma, Pennsylvania, South Carolina, South Dakota, Tennessee, Texas, Utah, Virginia, Washington, West Virginia, Wisconsin, Wyoming. ^c^ Each bar in the figure represents a 4-week sum of cigarette sales, averaged across products and across states within each state group.

Before the Massachusetts law, cigarette sales trends in border states were similar to non-border states (Figs 1 and 2). After the law, the unadjusted average of 4-week pack sales per 1000 people did not significantly change in border states for menthol, non-flavored, or overall cigarettes (Table 2). There was also no significant difference in unadjusted sales changes between border and non-border states.

**Table 2:** Cigarette sales^a^ of border versus non-border states, before and after Massachusetts law prohibiting flavored tobacco products sales.

	Average Four-Week Sales of Packs Per 1000 Persons (95% CI)			
	Menthol	Nonflavored	Overall	Sample
**Border States** ^b^				
*Before the law* ^c^	670.83 (559.29 to 782.37)	1876.11 (1369.83 to 2382.38)	2547.68 (1964.28 to 3131.09)	20
*After the law* ^d^	845.24 (742.88 to 947.59)	1924.97 (1668.13 to 2181.81)	2770.57 (2427.87 to 3113.27)	84
*Difference*	174.41 (-41.62 to 390.43)	48.86 (-526.19 to 623.91)	222.89 (-528.59 to 974.37)	NA
*P value*	0.11	0.87	0.56	NA
**Non-border States** ^e^				
*Before the law*	947.05 (883.29 to 1010.82)	2060.98 (1914.53 to 2207.42)	3011.35 (2829.14 to 3193.55)	140
*After the law*	921.99 (892.49 to 951.49)	2023.64 (1952.12 to 2095.17)	2949.20 (2860.89 to 3037.51)	588
*Difference*	-25.06 (-92.94 to 42.81)	-37.33 (-200.13 to 125.47)	-62.15 (-263.45 to 139.15)	NA
*P value*	0.47	0.65	0.54	NA
**Difference-in-differences Estimate**				
*Unadjusted* ^f^	199.47 (-61.27 to 460.21)	86.19 (-47.53 to 219.91)	285.04 (-102.64 to 672.71)	NA
*P value*	0.13	0.20	0.14	NA
*Adjusted* ^g^	184.42 (-79.88 to 448.72)	66.41 (-104.69 to 237.51)	250.81 (-173.78 to 675.40)	NA
*P value*	0.17	0.43	0.24	NA
**Sensitivity Analyses-Adjusted Difference-in-differences**				
*Including States with Local Bans*	148.91 (-72.54 to 370.37)	70.61 (-73.49 to 214.72)	219.33 (-136.54 to 575.19)	988
*P value*	0.18	0.33	0.22	NA
*Including the period 2014 to 2021*	145.14 (-124.55 to 414.83)	-80.88 (-301.20 to 139.43)	60.98 (-352.22 to 474.18)	3,072
*P value*	0.28	0.46	0.77	NA

Abbreviation: NA = Not Applicable

^a^Retail sales data were obtained from Information Resources, Inc. (IRI) for convenience stores, gas stations, grocery stores, drugstores/pharmacies, mass merchandiser outlets, club stores, dollar stores, and military sales. Unit sales were standardized by one pack of 20 cigarettes.

^b^ The list of border states included New Hampshire, Connecticut, Vermont, and Rhode Island.

^c^ In June 2020, Massachusetts implemented a law prohibiting the sale of all flavored tobacco products, including menthol cigarettes. The before-the-law period included January 2020 through May 2020.

^d^The after-the-law period included June 2020 to December 2021.

^e^The list of non-border states included the following 28 states: Alabama, Arizona, Arkansas, Florida, Georgia, Indiana, Iowa, Kentucky, Louisiana, Maryland, Michigan, Missouri, Nevada, North Carolina, North Dakota, Ohio, Oklahoma, Pennsylvania, South Carolina, South Dakota, Tennessee, Texas, Utah, Virginia, Washington, West Virginia, Wisconsin, Wyoming.

^f^The unadjusted difference in differences were obtained using linear regression models and an indicator for border states during June 2020 to December 2021. Standard errors were clustered within states. Unadjusted difference in differences could also be obtained by subtracting the unadjusted difference in non-border states from the unadjusted difference in border states.

^g^The adjusted difference in differences were obtained using linear regression models and an indicator for border states during June 2020 to December 2021. All models controlled for state and time fixed effects, real price per pack, tobacco control policies (cigarette taxes, smoke-free air laws, tobacco control funding), COVID-19 cases and deaths per 1,000 population and related statewide closure, and sociodemographic factors (population by race/ethnicity, age group, annual household income, and monthly unemployment rates). Standard errors were clustered within states.

The adjusted change in menthol cigarette sales in bordering states was not statistically different from the change in non-border states (DID = 184.42 packs per 1000 people; *P* = 0.17). Similarly, there were no statistically significant differences between border and non-border states in the adjusted changes in non-flavored cigarette sales (DID = 66.41; *P* = 0.43) or total sales (DID = 250.81; *P* = 0.24). These results persisted after including states with local menthol cigarette ban and periods before 2020.

## Discussion

Following implementation of a statewide law prohibiting flavored tobacco product sales in Massachusetts, unadjusted sales of menthol, non-flavored, and overall cigarettes trended upward in border states, however, unadjusted sales did not significantly change, or differ from sales patterns in non-border states during this period. This finding persisted after accounting for product prices, tobacco control policies, the COVID-19 pandemic, sociodemographic factors, and fixed effects.

Prior reports have found conflicting results related to changes in cigarette sales in border states following the Massachusetts law [3, 4]. However, these reports did not assess the statistical significance of sales trends or account for potential confounding factors, such as e-cigarette flavor polices, the COVID-19 pandemic, and differences across states in sociodemographic factors. After addressing these issues, we found no impact of the Massachusetts law on cigarette sales in border states.

This analysis is subject to at least three limitations. First, the data did not include sales made online; however, online cigarette sales were considerably restricted after passage of the Prevent All Cigarette Trafficking Act of 2009. Approximately 20% of e-cigarette sales were online in 2021, with the majority of sales occurring in physical stores [15]. Second, the observed sales patterns might have been influenced by factors that were not accounted for in the analysis; however, the analysis did account for a variety of potential confounding factors, including product prices, tobacco control policies, the COVID-19 pandemic, and sociodemographic factors. Finally, some exclusion criteria were applied to the data related to time period and assessed states; however, sensitivity analyses including the six excluded states with local menthol policies and periods before 2020 yielded similar results.

Flavors, including menthol, mask the harshness of tobacco and increase appeal, particularly among young people [16]. Menthol cigarettes also contribute to tobacco-related health disparities [17]. Laws prohibiting the sale of flavored tobacco products, including menthol, prevent and reduce access to these products [18], and had no statistically significant impact on cross-border sales in neighboring states where menthol cigarettes are sold.
